# Evaluating Modern Techniques for the Extraction and Characterisation of Sunflower (*Hellianthus annus* L.) Seeds Phenolics

**DOI:** 10.3390/antiox6030046

**Published:** 2017-06-24

**Authors:** Panagiotis Zoumpoulakis, Vassilia J. Sinanoglou, Eleni Siapi, George Heropoulos, Charalampos Proestos

**Affiliations:** 1 Institute of Biology, Medicinal Chemistry and Biotechnology (IBMCB), National Hellenic Research Foundation (NHRF), Vas. Constantinou Ave. 48, 11635 Athens, Greece; pzoump@eie.gr (P.Z.); esiapi@eie.gr (E.S.); gherop@eie.gr (G.H.); 2 Laboratory of Chemistry, Analysis & Design of Food Processes, Instrumental Food Analysis, Department of Food Technology, Technological Educational Institution of Athens, 12210 Athens, Greece; vsina@teiath.gr; 3 Food Chemistry Lab, Chemistry Department, National and Kapodistrian University of Athens, Panepistimiopolis, 15771 Athens, Greece

**Keywords:** phenolic compounds, sunflower, antioxidant capacity, high energy techniques, UHPLC-ESI-MS

## Abstract

Recently there is a great interest in using high energy techniques (HET) which involve microwave or ultrasound-assisted extraction (MAE and UAE) for isolation of natural bioactive compounds from plant foods. Such bioactive compounds are phenolics which were determined from sunflower (*Helianthus annuus* L.) kernels and hulls (defatted) utilising two different high energy extraction techniques, ultrasound and microwave assisted solvent extraction. All samples were characterised by ultra-high-performance liquid chromatography-electrospray ionization-mass spectrometry (UHPLC-ESI-MS). The effect of parameters such as the nature of the solvent, volume of solvent, temperature and time is discussed. It is proved that the techniques applied had reduced solvent consumption and shorter extraction times, and extraction yields of the analytes were equal to or to some extent higher than those obtained with conventional techniques. Total Phenolic Composition (TPC) of samples examined was studied by the Folin-Ciocalteu method and results were presented in μg gallic acid equivalents (GAE)/g dry extract. Kernels proved to have the higher amount of TPC while the press residues had shown comparable TPC results. The antioxidant activity of samples was spectrophotometrically determined by 2,2-Diphenyl-1-Picrylhydrazyl (DPPH) assay using Butylated hydroxyl toluene (BHT) as reference compound to compare with samples. Sunflower seeds (kernels) showed again the highest antiradical efficiency (AE) compared to hulls and press-residue extract. Afterwards, ferric reducing ability of plasma (FRAP) and trolox equivalent antioxidant capacity (TEAC) assays were used for measuring the antioxidant capacity of samples. Press residue, a by-product of sunflower oil extraction, contained phenolics as shown by UHPLC-ESI-MS analysis. Hence, later on these compounds can be possibly utilised by food or neutraceutical industries. Phenolic substances characterised in hulls, kernels, and press residue were phenolic acids, mainly chlorogenic, caffeic, cinnamic, 4-hydroxybenzoic and *p*-coumaric.

## 1. Introduction

Plant derived foods have always been utilised for their potent therapeutic properties and have been in use for many years for the treatment of diseases by traditional practitioners. Several substances from aromatic and pharmaceutical plants have been found to possess biological activities [1,2]. Searching for new natural bioactive substances, sunflower seeds, the fruits of the sunflower (*Helianthus annuus* L., Family: compositae) are important to scientists and researchers, initially due to the nutritive bioactive compounds they contain, but also due to their high antioxidant activity [3,4]. Sunflower seeds possess high nutritive value, being a good source of nutritional unsaturated fats, proteins, inorganic compounds, and phytochemicals. They have potent anti-inflammatory, anticancer, antioxidant, antihypertensive, analgesic, skin-protective, hypocholesterolemic, antibacterial activity and calming effects on nerves, muscles and blood vessels [5]. Today, two significant types of sunflower are cultivated: for production of oilseed and for non-oilseed production or for confectionery types [2]. However, less than ten percent of the total cultivation, production is accounted for confectionery type varieties, like pet foods and snacks [6]. Sunflower seeds have a high content of phenolic substances, mainly chlorogenic acid (CGA), which appears in the form of complexes or bound to proteins. The interaction of phenolic substances with sunflower proteins results in variation in stability and shelf life, and some changes in organoleptic properties [7].

The application of microwave assisted extraction (MAE) and ultrasound assisted extraction (UAE) to extraction of phenolic substances from sunflower kernels (defatted), hulls and press residue is presented. MAE and UAE are easy and rapid non-conventional extraction methods, applied to extract analytes out of solid matrices [8,9,10].

These two different extraction methodologies were compared both for total phenolic content and antiradical activity (AA). Furthermore, the phenolic profile of sunflower matrices was determined using the UHPLC-ESI-MS (ultra-high-performance liquid chromatography-electrospray ionization-mass spectrometry) technique. Also, four major variables: solvent polarity, solvent volume, temperature and extraction time were studied for their effects over antioxidant activity, TPC and phenolics composition from sunflower kernels, hulls and press residue extracts.

## 2. Materials and Methods

### 2.1. Reagents

Reagents: Folin-Ciocalteu, sodium persulfate, gallic acid (GA), BHT, 2,2′-azinobis-3-ethylbenzothiazoline-6-sulphonic acid, diammonium salt (ABTS), Trolox, ferrous chloride, and 2,2-diphenyl-1-picrylhydrazyl (DPPH), were purchased from Sigma-Aldrich (Athens, Greece); 2,4,6-Tris (2-pyridyl)-s-triazine (TPTZ) was purchased from Fluka Chemica (Athens, Greece). Dimethylsulfoxide (DMSO), (Merck KGaA, Darmstaadt, Germany) was used as a solvent. Water was obtained from a Milli-Q water purification system (TGI Pure Water Systems, Topway Global, Greenville, SC, USA). Lichrosolv hypergrade for LC-MS acetonitrile was supplied by Merck (Darmstadt, Germany). Water (18.2 MΩ) was from a Milli-Q water system (Millipore, Bedford, MA, USA). Acetic acid was from LGC Standards (Middlesex, UK). Eighteen standard phenolic compounds were used (gallic acid, protocatechuic acid, HydroxyTyrosol, 4-hydroxybenzoic acid, chlorogenic acid, gentisic acid, caffeic acid, vanillic acid, syringic acid, p-coumaric acid, rutin, ferulic acid, trans-m-hydroxycinnamic acid, o-coumaric acid, salicylic acid, luteolin, eriodictyol and cinnamic acid) purchased from Sigma Aldrich. 

### 2.2. Sunflower Samples

The plant matrices analysed consisted of sunflower kernels from dehulled sunflower seeds and hulls, as well as press residue from whole seeds donated by an industrial biofuel production company (Pavlos N. Pettas S.A., Patras, Greece). Three aliquots of 5 g of each matrix (hulls, kernels from whole seed and press residue) were defatted with 500 mL *n*-hexane in a Soxhlet apparatus and dried overnight at 20 °C. Then the defatted samples were freeze dried (Lyovac GT2, LH Leybold, Frenkendorf, Switzerland) and were homogenated to dried powder (20 mesh). Samples were kept at −20 °C until analysis. The whole procedure was performed three times (*N* = 9).

### 2.3. Preparation of Extracts

#### 2.3.1. UAE, by Water Bath

Methanolic and hydromethanolic (methanol: water 65:35 *v*:*v*) extracts were ultrasonicated as described below: Freeze dried samples (~1 g) were sonicated (40 W) for 15 min (Elmasonic S70, Elma, Singen, Germany), with 50 mL of each solvent at 30 °C for 1 h. Temperature was kept stable at 30 °C by adding cold water in the waterbath. Extract was then centrifuged at 4000 g for ten min, and filtered with paper (Whatman No. 4). Press residue was extracted with two more aliquots of each solvent (solvent total volume: l50 mL). Combined extract was evaporated at 40 °C until dry and redissolved in thirty percent of each solvent prior to further analysis [11].

#### 2.3.2. Microwave-Assisted Extraction (MAE)

Each lyophilised sample (~1 g) was inserted to a round bottom flask and 50 mL of each solvent (methanol and aqueous methanol (80:20 *v*:*v*) was added.

MAE was performed on a CEM (Discover SP-D, Matthews, NC, USA) open Vessel Microwave Digestion system at 50 W, 35 °C for 5 min. The extract was centrifuged at 4000 g for ten min, and filtered through paper (Whatman No. 4). Press residue was extracted with two more portions of each solvent (again total volume solvent: 150 mL). Combined extract was evaporated at 40 °C until dry and redissolved in thirty percent of each solvent prior to further analysis [12].

#### 2.3.3. TPC Determination

Total phenolics were quantified by the Folin-Ciocalteau (FC) method [13]. First, 800 μL of sodium carbonate (7.5 %, *w*:*v*) was added to 20 mL of each sample (ranging from 0.5 to 20 mg/mL), well mixed and left to stand for 2 min. Afterwards, 1 mL of FC reagent was added and the mixture was well shaken by vortex again. Samples were left in the dark for 30 min at room temperature, and then the absorbance was measured spectrophotometrically at 765 nm with a Cary 100 Conc. spectrophotometer. TPC was expressed in μg gallic acid equivalents (GAE)/mg dry extract, with the use of a standard curve ranging from 100–800 mg/L gallic acid (y = 0.6583x−0.0346, *R*^2^ = 0.9996). All determinations were performed three times at each separate content of standard and samples.

### 2.4. Determination of Antioxidant Activity (Applied Methods) 

#### 2.4.1. 1,1-Diphenyl-2-Picrylhydrazyl (DPPH) Assay

The ability of antioxidants to scavenge the stable organic nitrogen radical (DPPH^•^) is described [14]. In brief, 50 mL of various concentrations of sunflower seed extracts (ranging from 0.5 to 120 mg/mL) were added to 1.95 mL of DPPH prepared in methanol (0.1 mM) and mixed thoroughly. A control of DPPH only was also used. Absorbance was measured at 515 nm after 30 min incubation in the dark, at room temperature. Scavenging activity of samples was expressed as percentage of inhibition based on the following equation:

DPPH radical scavenging activity = ((*A_control_* − *A_sample_*)/*A_control_*) × 100, where *A_control_* absabsorbance value of control and *A_sample_* absabsorbance value of samples. IC_50_ of DPPH^•^ was determined from the plot of % inhibition vs sample concentration and presented in mg per mL. Antiradical efficiency was measured by the equation: AE=1/IC_50_ where AE stands for antiradical efficiency. Each analysis was performed a minimum of three times, and in triplicate, at each separate concentration. 

#### 2.4.2. Trolox Equivalent Antioxidant Capacity (TEAC) Assay

TEAC assay [15] was done after minor modifications [16]. Briefly, the ABTS^•+^stock solution was prepared by adding 2.5 mM potassium persulfate (Na_2_S_2_O_8_) to 7 mM ABTS (1:1 *v*:*v*), incubated at room temperature for 16 h prior to use. The stock ABTS^•+^ ethanol solution was measured at 734 nm. Ten μL of samples (range of 0.1–20 mg/mL) were added to 990 μL ABTS^•+^ solution. After 1 and 40 min, absorbance was checked after Trolox addition. Trolox stock solution (2.5 mM) was prepared in ethanol. A Cary 100 Con UV-spectrophotometer was used. TEAC linearity for the standard used was acceptable; concentrations ranged from 1–60 μM (*R*^2^ = 0.9899). Absorbance values of ABTS^•+^ were plotted vs Trolox concentration and the samples. TEAC values were calculated by dividing the regression coefficient of samples with the regression coefficient of Trolox. Results were presented in μmol Trolox/g dry extract. Each analysis was performed a minimum of three times, and in triplicate, at each separate concentration.

#### 2.4.3. Ferric Reducing Ability of Plasma (FRAP) Assay

FRAP assay determines the ability of compounds to reduce the non coloured [Fe^III^(TPTZ)_2_]^3+^ to the blue coloured [Fe^II^(TPTZ)_2_]^2+^ [17]. FRAP (88 mL) reagent was added to 55 μL acetate buffer and 60 μL of FeSO_4_·7H_2_O (ranged at 10–1000 μM) (standard) or of samples (extracts of sunflower seeds and BHT, 0.2–20 mg/mL), on 96-well plates. The absorbance was checked at 593 nm. FRAP had good linearity (for the range of 5–600 μM, y = 0.0036x + 0.0058, *R^2^* = 0.998) and sensitivity with LOD of 10μM FeSO_4_·7H_2_O. The antioxidant capacity of the extracts was expressed as μmol FeSO_4_·7H_2_O per g dry extract. All determinations were performed at least three times, and in triplicate, at each separate concentration of the standard and samples.

### 2.5. LC-MS Analyses

#### 2.5.1. Instrumentation—Analytical Conditions

LC measurements were performed by a Thermo Accela (UHPLC) by injecting a 3 µL sample from a 10 °C cooled tray directly onto the INTERCHIM column (2.1 mm × 100 mm, 1.7 µm, equilibrated in 5% solvent B (acetonitrile with 0.1% acetic acid) and 95% solvent A (0.2% acetic acid aqueous solution). Flow rate adjusted to 250 µL/min and compounds eluted by setting solvent B concentration from 5% to 53.8% for 13 min. Afterwards, column was cleaned with 95% solvent B (5 min) and equilibrated to 95% solvent A and 5% solvent B. Total run time, including cleaning and recalibration, was approximately 20 min. A Thermo Scientific LTQ Orbitrap Velos (Thermo Fisher Scientific, Bremen, Germany) hybrid mass spectrometer was in line to the U-HPLC via an HESI interface for LC/ESI-MS analysis. Negative ion mode with 4.5 kV voltage source was applied. The source heater temperature was 250 °C and the capillary temperature was 275 °C. Sheath gas and auxiliary gas flow rates were adjusted to 20 Arb and 10 Arb, respectively. ProteoMass LTQ-FT-Hybrid ESI Neg. Mode Cal Mix (SUPELCO, Bellefonte, PA, USA) was used for the daily calibrationing of instruments by testing the manufacturer’s mixture for calibration issues. All measurements were done in triplicate.

#### 2.5.2. IStatistics

Results are shown as means ± S.E. Statistics were performed via GraphPadInstat 3 software (GraphPadInstat Software, Inc., San Diego, CA, USA). Statistically significant differences (*p* < 0.05) were evaluated by the nonparametric Mann-Whitney test. 

## 3. Results and Discussion

### 3.1. Antioxidant Profile of Sunflower Hulls, Kernels and Press Residue

**Phenolic Content.** The TPC in methanol and aqueous methanol extracts of sunflower matrices, expressed as μg of GAEs/mg dry sample, are shown in Table 1. The TPC in aqueous-methanolic extracts was higher (*p* < 0.05) than in methanol extracts, obtained by UAE and MAE. Also, concerning UAE extracts, TPC of kernels was significantly higher (*p* < 0.05) than hull’s TPC for both used solvents. At the same time, the press residue originating from sunflower oil extracts proved to contain significant amounts of TPC in both solvents, but especially for aqueous methanol and for UAE method.

Results were similar to other research [18] when comparing kernel and hulls extracts from different sunflower seeds, indicating that the concentration of phenolic substances in kernels was much higher than in hulls using aqueous ethanol as the extraction solvent. Comparing results of the TPC in the two types of extraction, UAE increased TPC two times compared to MAE.

**Antioxidant Capacity of sunflower seed extracts.** The antioxidant activity of sunflower seed extracts (kernels, hulls and press residue) was determined in vitro by DPPH^•^ and ABTS^•+^ assays. The FRAP activity was also determined and compared to BHT (reference compound).

**Figure 1:**
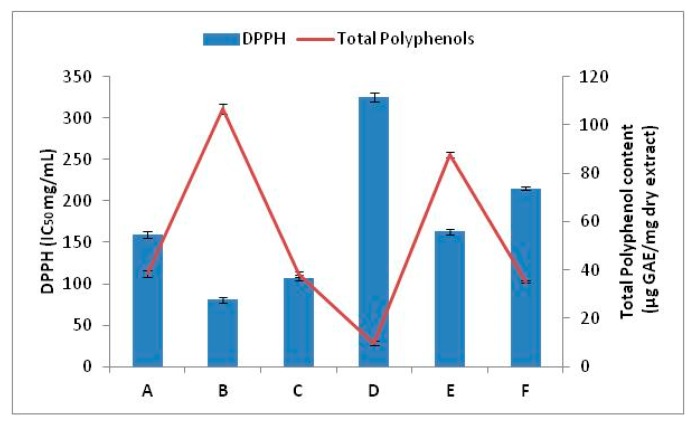
Effect of UAE and MAE extracts (aqueous methanol) on DPPH radical in comparison with Total Phenolic Composition (TPC). Results are expressed as mean ± S.E. Abbreviations used: A: kernels UAE, B: hulls, UAE, C: press residue, D: kernels MAE, E: hulls MAE, F: press residue MAE.

The IC_50_ and AE of sunflower seed extracts are presented in Table 2. It can be seen from the results that AE values of aqueous methanolic extracts were much higher than of the MeOH extract, irrespectively of the extraction type applied. By MAE extraction, IC_50_ values of sunflower seed extracts were reduced one to four times. The antioxidant capacity of sunflower seeds was measured by their ability to scavenge DPPH by similar studies [4,19]. There was a negative correlation between the scavenging ability on DPPH radical and TPC of both UAE and MAE extracts (MAE: *R^2^* = −0.6 and UAE: *R^2^* = −0.9), which shows that the scavenging activity of sunflower seed may be due to non-phenol substances, present in the sample extracts (e.g., amino acids, vitamins, peptides) (Figure 1). The MAE extracts correlation results was lower than the UAE, which shows that UAE may have an effect on both the antioxidant activity and phenolics in the extracts. Similar findings have been reported [20].

TEAC assay allows the evaluation of the antioxidant activity of both hydrophilic and lipophilic compounds present on the same sample. As shown in Table 3, sunflower seed extracts had lower (*p* < 0.05) TEAC values than BHT, at all times tested, irrespective of the extraction type applied. The ABTS^•+^ scavenging was better for aqueous methanol extracts in contrast to methanol extracts. Significant differences were not observed between the different extraction types applied, despite a small increase observed in the values of the aqueous methanol extract, obtained by UAE.

The antioxidant activity of the sunflower seed extracts was also tested by the FRAP assay, with FeSO_4_·7H_2_O being used as a reference standard. All extracts displayed lower antioxidant activity than BHT (151.3 ± 4.4 μmol FeSO_4_·7H_2_O/g dry extract), with FRAP values varying from 48.5–68.6 ± 3.0 μmol FeSO_4_·7H_2_O/g dry extract for UAE, and from 12.3–33.9 ± 0.3 μmol FeSO_4_·7H_2_O/g dry extract for MAE.

**Figure 2:**
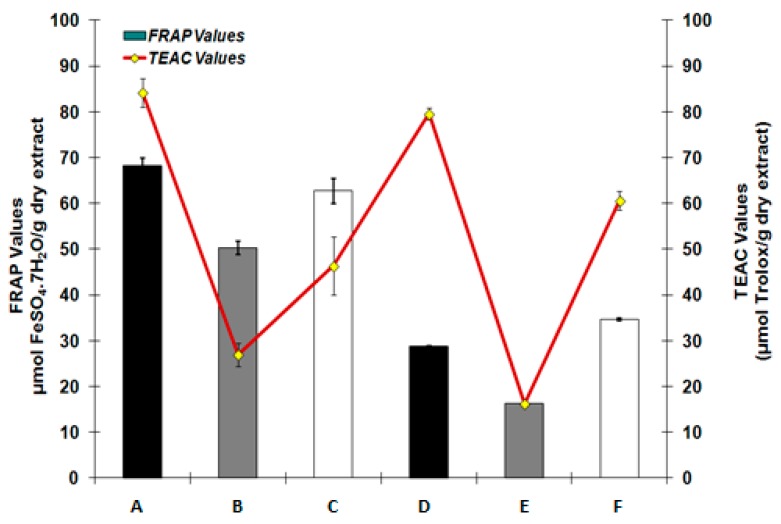
Correlation analysis between total antioxidant capacities measured by FRAP and TEAC assays. Abbreviations used: A: kernels UAE, B: hulls, UAE, C: press residue, D: kernels MAE, E: hulls MAE, F: press residue MAE.

Comparing the two extraction types, a significant reduction was detected in the FRAP values of the MAE samples. There was a major positive correlation between the antioxidant activity of FRAP and TEAC assay of sunflower seed extracts (*R^2^* = 0.7 and 0.9 for the MAE and UAE, respectively), showing that phenolic substances may play a role in antioxidant activity of sunflower seed extracts (kernels, hulls and press residue) (Figure 2). This result is similar to other studies [21,22].

### 3.2. Phenolic Profile of Selected Extracts

**Figure 3:**
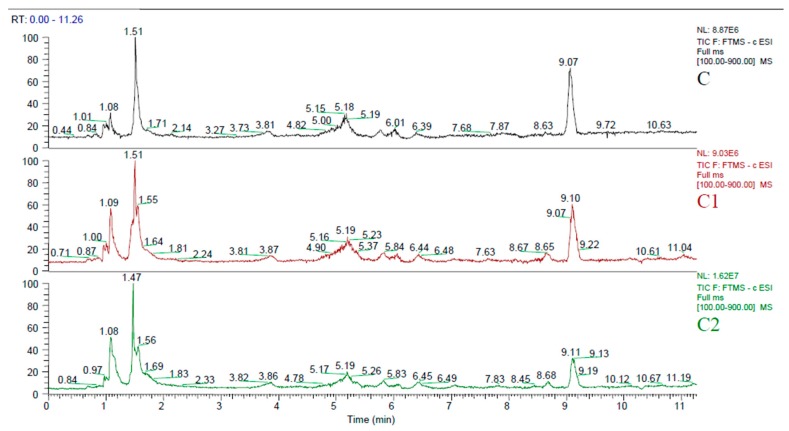
Total Ion Chromatogram (TIC) of kernels (C), hulls (C1), and press residue (C2) extracts.

**Figure 4:**
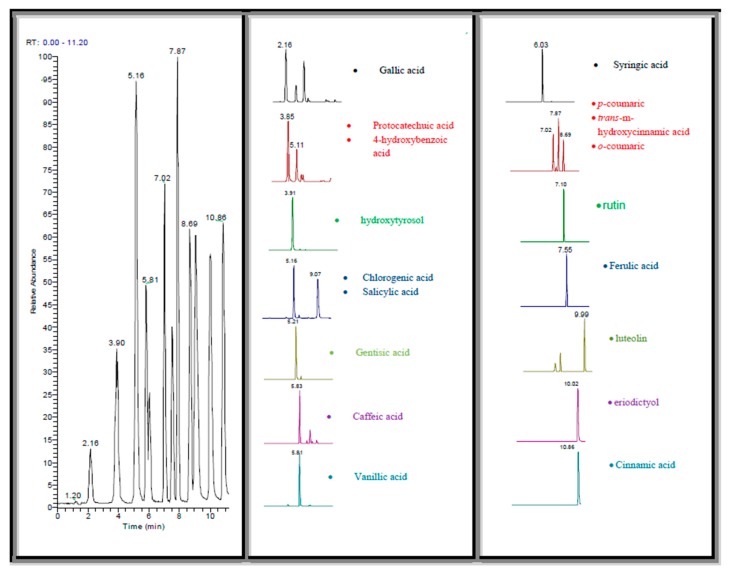
Retention times and accurate masses observed for four selected phenolic compounds in the kernels (C), hulls (C1), and press residue (C2) extracts compared to retention time and accurate mass of standard compounds.

LC-MS analysis was performed in order to characterise the main phenolic substances in UAE extracts of kernels, hulls and press residue. A mixture of 18 known phenolic compounds was created producing a standard mixture solution of 10ppm concentration which was subjected to LC-MS/MS. High resolution on an Orbitrap-based mass spectrometer can resolve phenolic compounds differing in mass by as little as 5 ppm, providing high selectivity. The combination of accurate mass and elution time provides confident confirmation of targets in complex mixtures. Total ion current of the three extracts is provided in Figure 3 showing similar patterns. By comparing the retention times and mass spectra obtained from standards and samples (Table 4, Figure 4), 15 compounds were identified in kernels, 16 in hulls and 18 in press residue extracts. Phenolic acids such as, chlorogenic, caffeic, salicylic and *4*-hydroxybenzoic were observed with higher intensity peaks in all three extracts. Vanillic acid was also observed at higher intensity but only in hulls and press residue. Finally, gallic acid was observed at higher intensity peak in kernels compared to hulls and press residue.

## 4. Conclusions

Results from this research proved that all extracts exerted good antioxidant activities. Higher antioxidant activities were shown for the aqueous methanolic extracts studied regarding the DPPH and TPC method, and for the methanolic extracts regarding the other two methods used (TEAC and FRAP). The extraction methods used had an effect on the antioxidant activities of the sunflower seed extracts, mainly for their concentration in TPC, which was highly increased in the UAE extracts. To our knowledge, there is little information about similar comparative studies to get info on high energy extraction techniques and their effects on the antioxidant activity of the extract. Ultrasound is one of the emerging technologies that were developed to minimise processing time and cost. MAE is a novel and green extraction method that can offer high reproducibility in shorter time, simplified manipulation, reduced solvent consumption and lower energy input without decreasing the extraction yield of the target compounds. Generally, the good antioxidant properties of sunflower seed (mainly kernel) highlights the nutraceutical and nutritional properties of this plant-derived food. Last but not least, the press residue coming from sunflower oil extract proved to have phenolics and could possibly be utilised by food industries for obtaining bioactive compounds such as phenolic compounds.

## Figures and Tables

**Table 1:** TPC results* by different solvents and extraction types Microwave Assisted Extraction (MAE) versus Ultrasound Assisted Extraction (UAE).

Lyophilised Samples/Extraction Type	Kernels		Hulls		Press Residue	
	Aq.MeOH	MeOH	Aq.MeOH	MeOH	Aq.MeOH	MeOH
MAE	160.0 ± 30.0 ^b,c^	80.7 ± 4.5 ^a,c^	80.0 ± 15.0 ^b,c^	35.5 ± 2.5 ^a,c^	107.4 ± 3.2 ^a,b^	46.4 ± 3.8 ^a,b^
UAE	325.5 ± 23.2 ^c^	163.0 ± 19.2 ^c,†^	165.5 ± 9.1 ^c^	72.0 ± 11.2 ^c,†^	215.0 ± 94.3 ^a,b,†^	93.0 ± 4.2 ^a,b,†^

* Results are in (μg GAE/mg dry extract, mean ± SE). Significant differences (*p* < 0.05) are shown by ^a,b,c^ letters in the same column; (^†^) shows statistically significant values for the two extraction methods (*p* < 0.05). MAE stands for Microwave Assisted Extraction and UAE stands for Ultrasound Assisted Extraction.

**Table 2:** IC_50_* and antiradical efficiency of sunflower seed extracts (kernels, hulls and press residue) in different extraction types and solvents (UAE versus MAE).

**Lyophilised Samples/Extraction Type**	**Kernels Aq.MeOH Extract ^a^**		**Kernels MeOH Extract ^b^**		**Press Residue Aq.MeOH Extract ^c^**	
	**IC_50_**	**AE**	**IC_50_**	**AE**	**IC_50_**	**AE**
MAE extraction	38.5 ± 5.0	0.030	106.6 ± 3.4	0.010	36.2 ± 1.6	0.030
UAE extraction	9.8 ± 1.1	0.121	87.6 ± 1.4	0.014	35.2 ± 1.3	0.022
**Lyophilised Samples/Extraction Type**	**Hulls Aq.MeOH Extract ^a^**		**Hulls MeOH Extract ^b^**		**Press Residue MeOH Extract ^c^**	
	**IC_50_**	**AE**	**IC_50_**	**AE**	**IC_50_**	**AE**
MAE extraction	79.5 ± 0.7	0.015	123.1 ± 0.9	0.010	38.8±1.1	0.010
UAE extraction	36.8 ± 1.5	0.030	126.6 ± 0.8	0.010	40.5±5.0	0.010

Values are mean ± S.E calculated from three different experiments performed in duplicate. Significant differences (*p* < 0.05) are shown by ^a,b,c^ letters in the same column; Statistical analysis was performed with GraphPad Instat 3 software, using the non-parametric Mann-Whitney test. Abbreviations used: MAE: Microwave Assisted Extraction; UAE: Ultrasound Assisted Extraction. * IC_50_ values are expressed in mg/mL.

**Table 3:** Results* from TEAC assay (results expressed in μmol Trolox per g dry extract) for sunflower seed extracts (kernels, hulls and press residue) in different extraction types and solvents (UAE versus MAE), and at time intervals of 1–40min.

Samples/Extraction Type		Aq.MeOH Extract ^a^				MeOH Extract ^b^			
		1	10	20	40	1	10	20	40
UAE	BHT^(^*^)^	52.5 ± 3.1	63.9 ± 2.9	72.2 ± 3.1 ^b,c^	91.2 ± 3.7 ^b,c^	41.3 ± 2.0	52.6 ± 2.5	61.3 ± 2.1 ^a,b^	80.9 ± 2.2 ^a,b^
	Kernels	31.6 ± 2.5 ^b,c^	41.5 ± 1.7 ^c^	42.7 ± 5.3 ^b^	50.8 ± 1.0 ^b^	17.7 ± 4.1^a,b^	22.5 ± 6.5 ^a,b^	22.4 ± 5.4	29.3 ± 9.5
	Hulls	6.4 ± 1.5 ^b,c^	12.5 ± 2.7 ^c^	13.5 ± 4.3 ^b^	21.6 ± 1.1 ^b^	3.1 ± 0.5 ^a,b^	6.5 ± 0.8 ^a,b^	6.6 ± 0.4	13.3 ± 1.1
MAE	BHT^(^*^)^	49.5 ± 3.2	61.9 ± 3.2	70.2 ± 2.1 ^b,c^	89.1 ± 3.4 ^b,c^	38.6 ± 2.5	48.9 ± 2.9	60.3 ± 1.9 ^a,b^	78.2 ± 2.6 ^a,b^
	Kernels	30.4 ± 2.4 ^b,c^	40.3 ± 1.8 ^c^	41.5 ± 4.9 ^b^	48.8 ± 1.5 ^b^	16.8 ± 3.9 ^a,b^	20.5 ± 6.2 ^a,b^	21.1 ± 4.7	28.4 ± 9.1
	Hulls	5.9 ± 1.5 ^b,c^	11.9 ± 2.5 ^c^	12.9 ± 4.1 ^b^	20.9 ± 1.3 ^b^	2.8 ± 0.6 ^a,b^	5.7 ± 0.7 ^a,b^	5.8 ± 0.5	12.7 ± 1.3

* Results are presented as mean ± S.E (four different experiments done in triplicate. Significant differences (*p* < 0.05) are shown by ^a,b,c^ letters in the same column. MAE stands for Microwave Assisted Extraction and UAE stands for Ultrasound Assisted Extraction.

**Table 4:** m/z [M−H]^−^, retention times (RT) and mass deviation (mDa) of the 18 standard phenolic substances in the kernels (C), hulls (C1), and press residue (C2) of the analysed samples.

Standards	[M−H]^−^	RT				Delta (mDa)		
Compound Name	m/z	standards	C	C1	C2	C	C1	C2
gallic acid	169.014	2.16	2.16	2.11	2.17	0.1	0.2	0.1
protocatechuic acid	153.019	3.85	3.83	3.83	3.84	0.1	0.0	−0.0
hydroxyTyrosol	153.056	3.91			4.14			−0.0
4-Hydroxybenzoic acid	137.024	5.11	5.02	5.04	5.03	−0.1	−0.1	−0.1
chlorogenic acid	353.088	5.16	5.18	5.19	5.19	−1.5	−1.0	−1.5
gentisic acid	153.019	5.21		5.13	5.2		0.1	−0.4
caffeic acid	179.035	5.83	5.77	5.83	5.83	−0.2	−0.2	−0.2
vanillic acid	167.035	5.81	5.76	5.82	5.81	−0.1	−0.1	−0.0
syringic acid	197.046	6.03	5.95	6.02	6.03	−0.8	−0.5	−0.2
*p*-coumaric acid	163.04	7.02	6.98	7.01	7.02	−0.1	−0.1	−0.1
Rutin	609.146	7.10		7.08	7.1		−1.4	−0.5
ferulic acid	193.051	7.55	7.51	7.54	7.56	−0.2	−0.3	−0.2
Trans-m-hydroxycinnamic acid	163.04	7.87		7.92	7.93		−0.2	−0.1
*o*-Coumaric acid	163.04	8.69			8.65			−0.2
salicylic acid	137.024	9.07	9.07	9.12	9.19	0.1	−0.1	0.0
Luteolin	285.041	9.99	9.96	9.99	10.01	−0.5	−0.8	−0.9
Eriodictyol	287.056	10.02	9.95	9.99	10	−0.5	−0.8	−0.7
cinnamic acid	147.045	10.86	10.81	10.86	10.88	0.2	0.2	0.2
